# Case Report: A Case of Neuroendocrine Carcinoma of the Endometrium with Deficient DNA Mismatch Repair Had Achieved Clinical Complete Response after Combination Therapy

**DOI:** 10.32604/or.2026.071213

**Published:** 2026-04-22

**Authors:** Xie Ding, Xinfeng Liu, Cheng Dai, Xiaoxiao Wang, Zhaopeng Zheng

**Affiliations:** 1 The Second Clinical College, Zunyi Medical University, Zunyi, China; 2 Department of Oncology, The People’s Hospital of QianNan, Duyun, China; 3 Department of Oncology, Guizhou Provincial People’s Hospital, Guiyang, China; 4 Department of Radiology, Guizhou Provincial People’s Hospital, Guiyang, China; 5 Department of Gynecology, Guizhou Provincial People’s Hospital, Guiyang, China

**Keywords:** Neuroendocrine carcinoma of the endometrium (NECE), DNA mismatch repair deficiency (dMMR), programmed death-1 (PD-1) inhibitor, camrelizumab, combination chemotherapy, adjuvant radiotherapy, case report

## Abstract

**Background:**

Immunotherapy has markedly reshaped the therapeutic landscape for patients with postoperative progression and metastasis. As a programmed death-1 (PD-1) inhibitor, camrelizumab has been proven to exhibit both efficacy and safety in the treatment of advanced dMMR solid tumors.

**Case Description:**

A 58-year-old female patient with neuroendocrine carcinoma of the endometrium (NECE) who was treated with camrelizumab coupled with chemotherapy, subsequent maintenance monotherapy with camrelizumab, and adjuvant pelvic local radiotherapy. Up to December 2024, the patient has survived 28 months since treatment, with 26 months free from disease progression, and the assessment indicated a status of clinical complete response (cCR).

**Conclusion:**

The combined regimen achieves notable efficacy and is free of unexpected adverse effects, with only a radiotherapy-related pelvic insufficiency fracture (PIF) report.

## 1 Introduction

Endometrial cancer is the second most prevalent gynecologic malignancy across the globe [1]. Progress in genomic profiling has recently confirmed that approximately 20%–30% of endometrial cancer cases feature DNA mismatch repair deficiency (dMMR) [2]. Yet, endometrial neuroendocrine carcinomas are exceedingly rare, highly invasive malignancies characterized by poor prognostic outcomes. Programmed death-1 (PD-1) inhibitors show significant efficacy in treating mismatch repair deficiency, according to the National Comprehensive Cancer Network (NCCN) and Chinese Society of Clinical Oncology (CSCO) guidelines [3,4]. We herein report a case of a middle-aged female patient diagnosed with neuroendocrine carcinoma of the endometrium (NECE), who experienced recurrence and lymph node metastasis after surgery and the first chemotherapy. The patient reached complete relief following the combination treatment of 5 cycles of camrelizumab and EP (etoposide + cisplatin) combined regimen, 25 cycles of camrelizumab single-drug maintenance immunotherapy, and 26 fractions of pelvic local radiotherapy.

This study was approved by the ethics committee of Guizhou Provincial People’s Hospital, with the reference number: 2025-130 (Supplementary Material S1). The handwritten informed consent was obtained from the patient (Supplementary Material S2). Besides, this study was prepared according to the CARE case report guideline [5], and a CARE checklist was provided (Supplementary Material S3). Please see Supplementary Material for more details.

## 2 Case Description

A 58-year-old postmenopausal woman (BMI 22, G4P4) with no significant past medical history presented to the local hospital with three months of irregular vaginal bleeding without an obvious cause. Further history-taking revealed no family history of malignancy. Pelvic magnetic resonance imaging (MRI) revealed a space-occupying lesion in the uterine cavity. The diagnosis suggested a high possibility of endometrial cancer, and surgery was recommended. The patient was then admitted to Guizhou Provincial People’s Hospital for further treatment.

**Figure 1:**
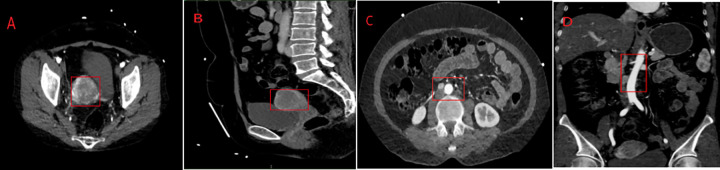
Preoperative imaging. Pelvis CT showed (**A**, **B**) thickening and enhancement of the endometrium. Abdominal enhanced CT scan (**C**, **D**) showed increased retroperitoneal and bilateral perivascular lymph nodes with partial enlargement. Red box: Indicates lesion and increased lymph nodes.

On July 10, 2022, abdominal and pelvic enhanced Computed Tomography (CT) showed thickening and enhancement of the endometrium (Fig. 1A,B), presenting features of endometrial cancer, along with increased retroperitoneal and bilateral perivascular lymph nodes with partial enlargement (Fig. 1C,D).

**Figure 2:**
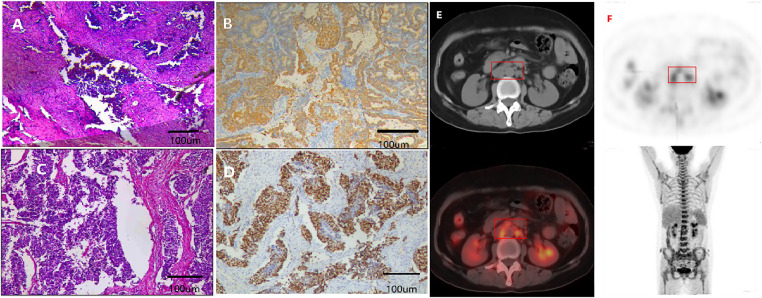
Postoperative pathological examination (H&E, IHC 100×; scale bar = 100 μm) and PET-CT scan. Hysteroscopic endometrial biopsy revealed well-differentiated endometrioid carcinoma and neuroendocrine carcinoma (**A**, **B**). Hematoxylin and eosin (H&E) of post-operation biopsy confirmed well-differentiated endometrioid carcinoma, approximately 5%; high-grade neuroendocrine carcinoma, approximately 95% (**C**). IHC showed positivity for MLH1, MSH2, and PMS2, and negativity for MSH6 (**D**). PET/CT showed multiple lymph node metastases in the retroperitoneum and right iliac blood vessels after surgery (**E**, **F**). Red box: Increased lymph nodes.

**Figure 3:**
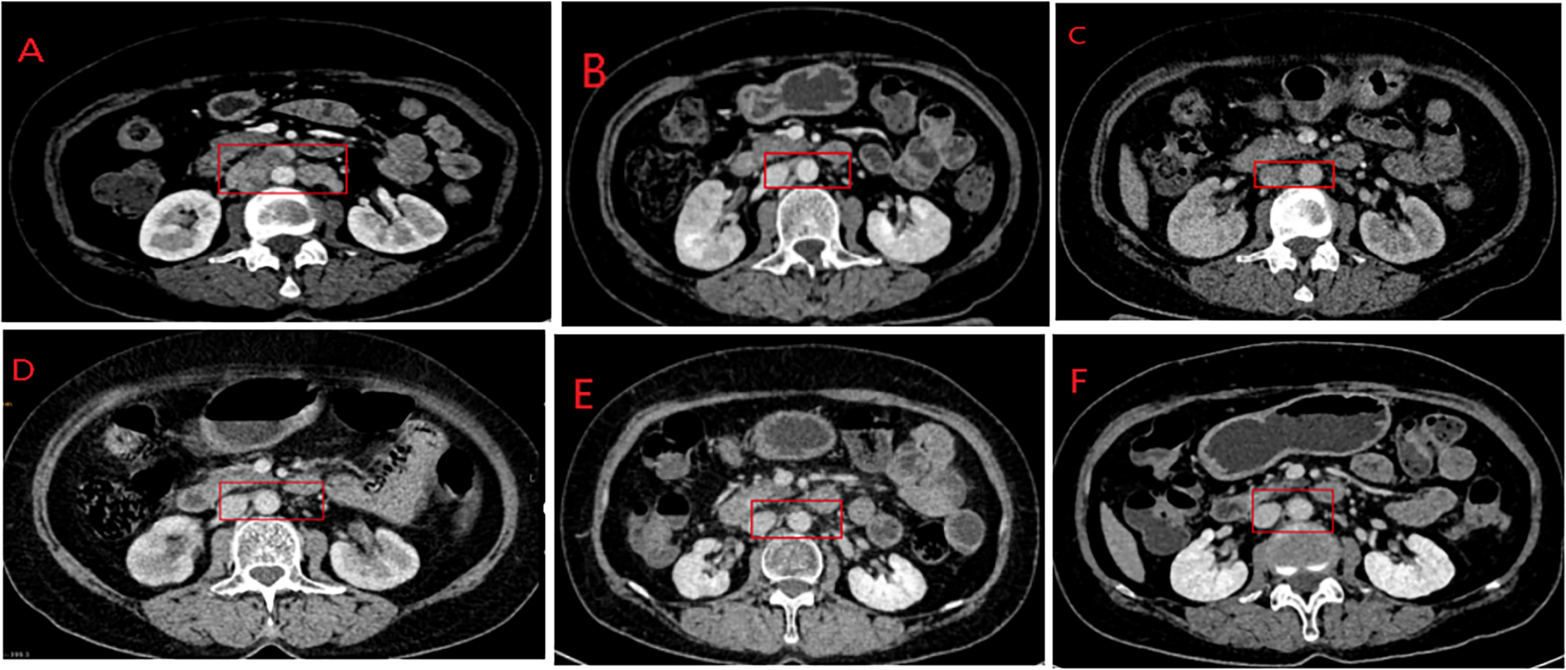
Abdominal CT scans of the patient. (**A**) CT showed multiple enlarged lymph nodes in the retroperitoneum, the largest one of which measures approximately 22 mm; it was regarded as the target lesion. (**B**) After two cycles of the combination of EP and camrelizumab, CT demonstrated shrunken the multiple lymph nodes in the retroperitoneum, the larger one has a short-axis of approximately 7 mm. (**C**) 5 cycles of camrelizumab + EP, CT showed that retroperitoneal lymph nodes further size reduction, the maximum diameter was approximately 6 mm. (**D**) Monotherapy with camrelizumab for sustained immunotherapy, CT scan revealed that the largest short axis diameter of the retroperitoneal lymph node to be 5 mm. (**E**) After 15 cycles of camrelizumab, the retroperitoneal lymph nodes remain at 5 mm in size. (**F**) Abdominal CT showed the target lesion was maintained at 5 mm in size. Red box: Retroperitoneal lymph nodes.

The hysteroscopic endometrial biopsy confirmed the diagnosis of malignancy, with histological types showing both well-differentiated endometrioid carcinoma and neuroendocrine carcinoma (Fig. 2A,B). On August 4, 2022, the patient underwent a laparoscopic total hysterectomy, bilateral salpingo-oophorectomy, and pelvic and paraaortic lymphadenectomy without intraoperative complications. However, the tumor tissue was not completely removed during the operation, as the para-abdominal aortic lymph nodes were densely adherent to the inferior vena cava and abdominal aorta. Postoperative pathology revealed infiltrating carcinoma of the uterus (well-differentiated endometrioid carcinoma (~5%), and high-grade neuroendocrine carcinoma (~95%)), breach through the serosal layer, involving both ovaries, bilateral parametrial, and cervical stroma. Immunohistochemistry results showed cervical canal tissue: CgA (+). Endometrial-like carcinoma component: CKpan (+), Vimentin (+), CK7 (+), Pax-8 (+), ER (95%+), PR (70%+), EMA (+), P16 (patchy+), P53 (diffuse strong+), CD10 (−), HNF1-β (−), CK20 (−), NapsinA (−), WT-1 (−), CD56 (−), CgA (−), Syn (−), Ki67 (40%+). Neuroendocrine carcinoma component: CKpan (+), CK7 (+), CD56 (+), CgA (+), Syn (+), EMA (+), P16 (diffusely strong+), P53 (weak+ in some cells), Vimentin (−), ER (−), PR (5%+), CD10 (−), Pax-8 (–), HNF1-β (–), CK20 (–), NapsinA (–), WT-1 (–), Ki67 (90%+) (Fig. 2C). The size of the tumor was approximately 6.5 × 4.5 × 4 cm. In addition, vascular and nerve invasions, as well as pelvic and para-abdominal aortic lymph nodes metastasis, were observed. According to the postoperative pathological examination, the patient was diagnosed with NECE IVB. The patient subsequently received the first cycle of the EP regimen (etoposide + cisplatin) in the surgical department on August 13, 2022. After the surgery and first cycle of chemotherapy, the patient experienced high fever and elevated white blood cells in the urine, and blood culture confirmed the septic shock caused by *Escherichia coli* infection. After consultation with the infectious and respiratory departments, the patient was diagnosed with a urinary tract infection and sepsis. The patient was then transferred to the intensive care unit for anti-infection and symptomatic support treatment to alleviate the symptoms. Immunohistochemistry (IHC) of the Ventana testing platform showed 10% PD-L1 expression (Tumor Cell/Tumor cell Proportion Score [TC/TPS]), and tested positivity for MLH1, MSH2, and PMS2, but negativity for MSH6 (Fig. 2D). On August 25, 2022, positron emission tomography/computed tomography (PET/CT) showed multiple lymph node metastases in the retroperitoneum (adjacent to large blood vessels) and right iliac blood vessels (Fig. 2E,F). On September 14, abdominal CT showed multiple enlarged lymph nodes in the retroperitoneum, the largest one measuring approximately 22 mm, indicating the high possibility of metastasis and recurrence (Fig. 3A). Since the patient’s primary tumor has been surgically removed, the retroperitoneal lymph nodes are considered the target lesions for efficacy assessment.

Based on the IHC results and the patient’s financial circumstances, on September 14, 2022, following the patient’s approval, the therapeutic protocol was switched to camrelizumab (200 mg) in conjunction with the EP regimen. Based on a body-surface area of 1.46 m^2^, etoposide was dosed at 140 mg (Days 1–3) and cisplatin at 110 mg (administered intravenously over 3 consecutive days), corresponding to the standard doses of 100 mg/m^2^ and 80 mg/m^2^, respectively. The core rationale for administering cisplatin in divided doses is to reduce toxicity to normal tissues such as the kidneys and gastrointestinal tract while maintaining antitumor efficacy, thereby enhancing treatment safety and tolerability. The cycle will be repeated every 21 days. On October 26, 2022, a follow-up abdominal CT showed that after 2 cycles of combined treatment with camrelizumab and EP regimen, there was a massive reduction of retroperitoneal lymph nodes; the larger one had a short-axis of approximately 7 mm (Fig. 3B). The patient achieved a Partial Response (PR) after evaluation by the treatment team. Following the second cycle of treatment, the patient experienced third-degree myelosuppression and recurrent urinary tract infections, and the symptoms were relieved after G-CSF administration and antibiotic therapy, so the third cycle of treatment was delayed by 5 days. After 5 cycles of combination therapy, an abdominal CT scan on December 18, 2022, revealed further shrinkage of retroperitoneal lymph nodes, with the largest measuring approximately 6 mm in short diameter (Fig. 3C). The patient achieved a clinical complete response (cCR) after evaluation. Starting from January 2, 2023, we switched the treatment regimen to monotherapy with camrelizumab (200 mg, every three weeks) for sustained immunotherapy.

During the 9th cycle of immunotherapy, the local external-beam radiotherapy (Volumetric Modulated Arc Therapy, VMAT) of the pelvic lymph node drainage area was included from February 28 to April 1, 2023. The prescribed radiation therapy dose targeted the upper vaginal wall remnant, and the regional lymph node drainage area was 95% PTV = 4680cGy/180cGy/26F, with the dose for positive lymph node regions increased to 95% PGTVnd = 5980cGy/230cGy/26F. Detailed plans and Dose-Volume Histogram (DVH) are available in the supplementary materials (Supplementary Fig. S1). The patient’s condition remained stable during the radiotherapy period. Re-examinations were conducted in February 2023 (Fig. 3D), July 2023 (Fig. 3E), and June 2024 (Fig. 3F), all of which showed the largest short axis diameter of the retroperitoneal lymph node to be 5 mm. This persistent 5 mm shadow is likely no longer an active tumor cell but rather scar tissue formed after infiltration by immune cells. Imaging studies (CT) cannot distinguish between active tumors and fibrous scar tissue. More precise examinations, such as PET-CT, may show no metabolic activity at this site, but due to financial constraints, a follow-up PET-CT scan was not pursued. Following clinical assessment, the patient achieved a clinical complete response (cCR) to the treatment regimen.

**Figure 4:**
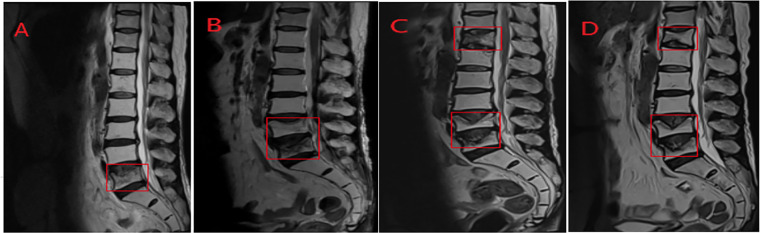
Lumbar Spine MRI scanning of the patient. (**A**) Compression Fracture of the L5 Vertebral Body. (**B**) Compression Fracture of the L4 and L5 Vertebral Bodies. (**C**) Compression Fracture of the T12, L4, and L5 Vertebral Bodies. (**D**) Bone destruction in the sacrum and the posterior part of bilateral iliac bones, and Compression Fracture of the T12, L4, and L5 Vertebral Bodies. Red box: Area of bone destruction.

**Figure 5:**
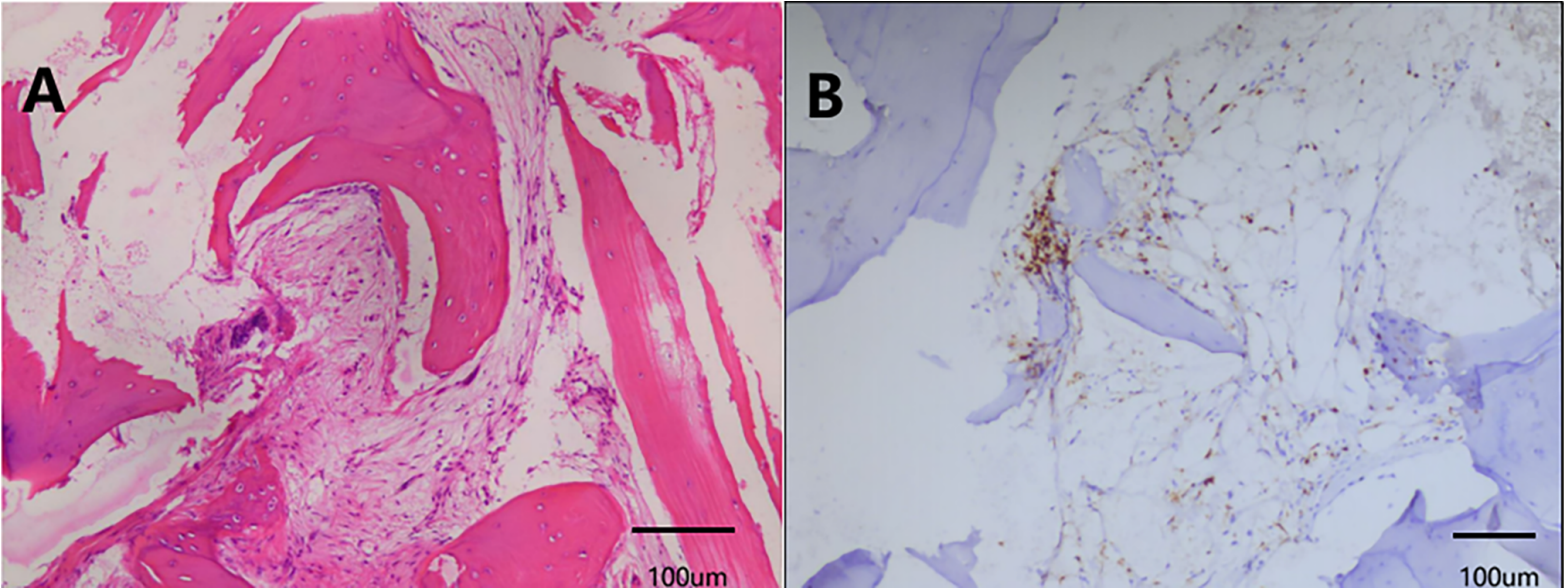
Bone puncture pathologic biopsy (H&E, IHC 100×; scale bar = 100 μm). (**A**) Hematoxylin and eosin (H&E) of the lesional tissue of the right ilium confirmed Trabecular bone fibrous tissue hyperplasia with sequestrum formation. (**B**) IHC confirmed Lymphocyte proliferation and the absence of tumor cells.

**Figure 6:**
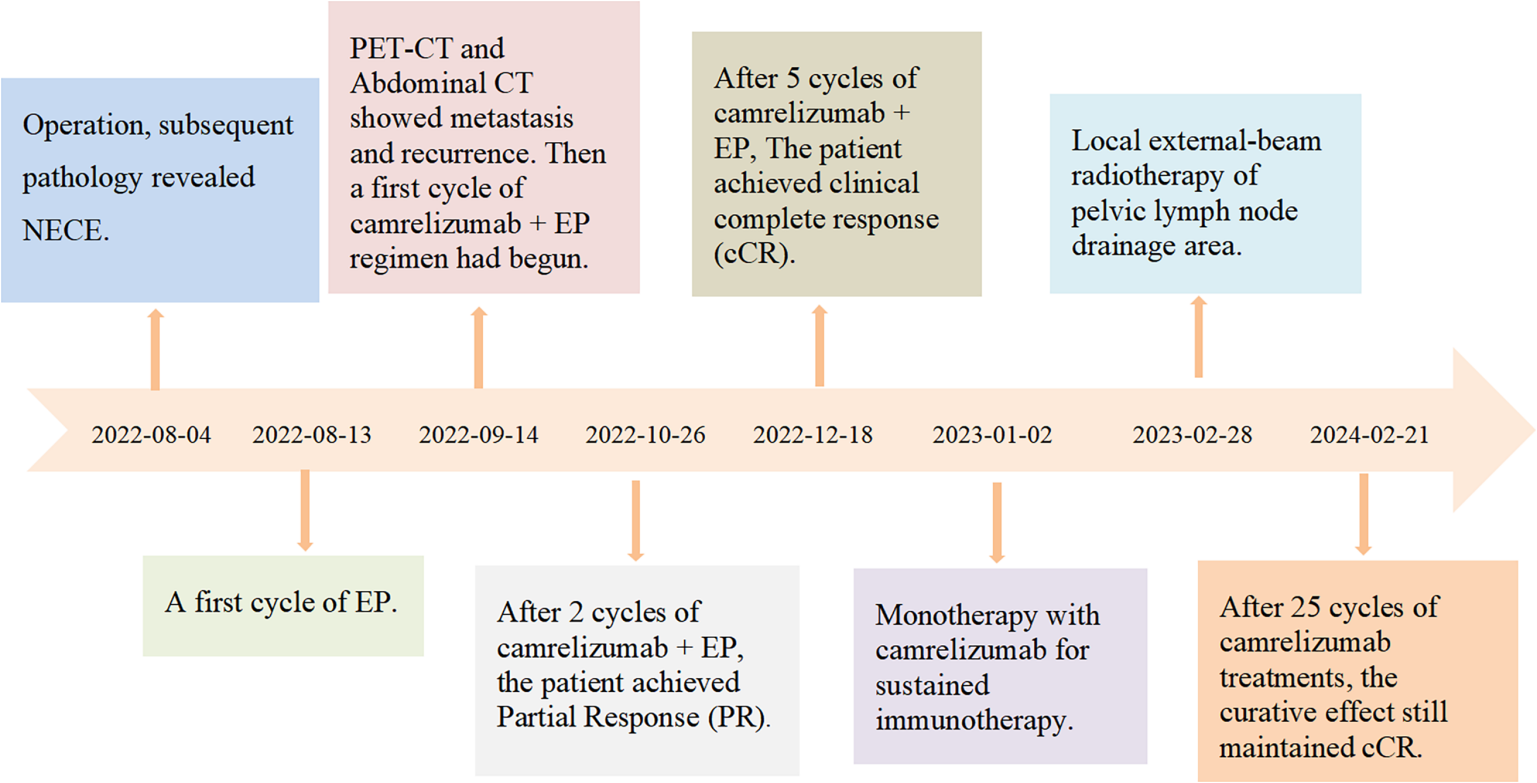
A timeline of key points in the patient’s treatment.

Upon completion of combined chemotherapy, the patient experienced only mild myelosuppression and no further urinary infections. The patient continued to receive 200 mg of camrelizumab every 3 weeks without additional adverse events. By September 2023, the patient received 18 cycles of immunotherapy using camrelizumab. However, on September 11, 2023, the patient reported lumbosacral pain, and the lumbar spine MR scan showed a vertebral compression fracture and degenerative disease of the lumbar spine (Fig. 4A), yet no definitive sign of bone metastasis was observed. Considering the patient’s age, we opted to continue with observation and symptomatic management, but the symptoms did not improve. Several subsequent lumbar spine MR scans revealed new fractures of the lumbar and thoracic vertebrae, along with bone destruction of the sacrum and ilium (Fig. 4B–D). The patient was then hospitalized for right hip pain in June 2024. The preliminary diagnoses suggested metastasis based on the symptoms and imaging reports; a bone puncture pathologic biopsy of the patient’s right iliac bone was performed to confirm the cause of the disease. Pathological results indicated the presence of a few lymphocytes and the absence of tumor cells (Fig. 5A,B), which ruled out the possibility of metastasis. The patient was advised to take calcium supplements and improve nutritional intake to prevent osteoporosis. Simultaneously, the maintenance therapy with camrelizumab proceeds. The treatment timeline and changes in the target lesion during treatment are shown in Fig. 6 and Table 1. Up until December 2024, the patient has received 25 cycles of camrelizumab treatment. From the time of recurrence, the patient has survived for 28 months. We will continue to follow up with changes in the patient’s condition.

**Table 1:** Changes in the target lesion over the course of treatment.

Treatment Duration and Cycle	Target Lesion Size (The Retroperitoneal Lymph Node)
Operation and first cycle of EP, on September 14, 2022	The larger one has a short-axis of approximately 22 mm
2 cycles of camrelizumab and EP regimen, on October 26, 2022	The larger one has a short-axis of approximately 7 mm
5 cycles of camrelizumab and EP regimen, on December 18, 2022	The larger one has a short-axis of approximately 6 mm
After 9 cycles of camrelizumab, in February 2023	The larger one has a short-axis of approximately 5 mm
After 15 cycles of camrelizumab, in July 2023	The larger one has a short-axis of approximately 5 mm.
The last follow-up examination was in June 2024	The larger one has a short-axis of approximately 5 mm

## 3 Discussion

Neuroendocrine carcinomas (NEC) usually occur in the gastrointestinal tract, pancreas, or lung [6,7], but are rare in the endometrium and cervix [8]. NECE is an extremely rare and aggressive malignant disease, characterized by early hematogenous and lymphatic metastases [9], which accounts for less than 1% of all endometrial cancers (EC) [10]. A previous retrospective study from the National Cancer Database (NCDB) showed that NECE had a worse prognosis than EC patients; the median survival was 17 months for NECE and 144 months for EC, and the 5-year survival was 38.3% for NECE vs. 68.8% for EC [9]. In a review of 25 NECE cases, all patients, especially those who presented with stage IVB disease, died of disease at 2–35 months after diagnosis (median, 9 months) [11]. No extensive studies or prospective clinical trials have been conducted to guide the therapy in NECE; thus, data are largely extrapolated from the treatment of NEC. The standard recommended first-line treatment of NEC is the platinum-etoposide chemotherapy regimen regardless of the location of the primary tumor site [12,13]. Accordingly, towards unresectable and metastatic NECE, etoposide + platin-based systemic chemotherapy remains the primary treatment, yet still lacks specific data due to its rare occurrence. The NCT 03043872 study recruited 269 patients with untreated extensive-stage small-cell lung cancer (ES-SCLC) to receive EP alone chemotherapy; the median overall survival (OS) was 10.3 months, with only 25% of patients alive at 18 months [14]. The TOPIC-NEC Phase 3 Randomized Clinical trial enrolled 84 patients with advanced NEC of the digestive system. Patients were treated with etoposide plus cisplatin (EP) chemotherapy; the result showed the median overall survival (OS) was 12.5 months and the median progression-free survival (PFS) was 5.6 months [15]. The above treatment results show that the overall efficiency and survival time of chemotherapy alone are of limited efficacy, suggesting the urgent need for the development of new treatment approaches through further prospective studies.

The exploration of new treatment plans has become a particular research focus to improve the survival status of patients with dMMR NECE. Previous research proposed the mismatch-repair dysfunction and microsatellite instability as the main characteristics of NECE [16]. A phase 2 study reported that pembrolizumab, a PD-1 inhibitor, is more responsive to mismatch repair-deficient tumors, and the result showed that patients with dMMR cancer who were treated with pembrolizumab had a better prognosis [17]. Moreover, dMMR cancers contain prominent lymphocyte infiltrates, which contribute to the better prognosis than mismatch repair-proficient (pMMR) [18]. A study by Le et al. enrolled 86 patients with advanced dMMR cancers across 12 different tumor types, including both NEC and EC, to evaluate the efficacy of pembrolizumab. The result showed that the disease control (measured as partial response + complete response + stable disease) was achieved in 66 (77%) of the 86 patients (95% CI, 66–85%) [19]. Moreover, another clinical trial, KEYNOTE 177, assigned 307 patients with previously untreated dMMR metastatic colorectal cancer in a 1:1 ratio, to receive pembrolizumab or chemotherapy, the result demonstrated that the PFS of pembrolizumab was 16.5 months (95% CI 5.4–38.1) twice as longer than the PFS of 8.2 months for chemotherapy (HR 0.59, 95% CI 0.45–0.79) [20]. The occurrence of treatment-related adverse events, as well as events of grade 3 or worse in the pembrolizumab group (22%), was lower compared with the chemotherapy group (66%). Simultaneously, pembrolizumab continuously demonstrates the anti-tumor activity [21]. Other PD-1 inhibitors, like dostarlimab, also gained promising results in the NCT02715284 study [22]. Among 129 patients with dMMR advanced EC who received dostarlimab, the median follow-up duration was 16.3 months, and the Objective Response Rate (ORR) was 43.5%, with 11 complete responses and 36 partial responses. In recent years, immunotherapy and chemotherapy combined treatments have shown excellent curative effects. An NRG-GY018 trial enrolled 816 patients with dMMR EC and pMMR EC, receiving pembrolizumab or placebo along with combination therapy with paclitaxel plus carboplatin. The result showed the PFS were 74% and 38% in the pembrolizumab and placebo group (hazard ratio for progression or death, 0.30; 95% confidence interval [CI], 0.19 to 0.48; *p* <0.001), respectively, with a 70% difference in relative risk [23]. In the phase 3 RATIONALE-312 study, patients with previously untreated ES-SCLC were randomly assigned to receive tislelizumab, a PD-1 inhibitor, or placebo, plus EP. The results demonstrated that both the OS and PFS were significantly improved in the tislelizumab versus placebo arm [24]. In summary, previous studies have shown that the combination of immunotherapy with chemotherapy is effective for dMMR patients, and the side effects are manageable. Therefore, we are also exploring the use of combination therapy in our case to further validate its efficacy.

Although the pembrolizumab significantly improves clinical outcomes in advanced/metastatic dMMR cancers, the cost for this antibody is high. To our delight, camrelizumab, the cheapest PD-1 inhibitor, was approved for several types of cancer in China after insurance reimbursement, which could be exciting for many patients in need. Although a lack of data from randomized studies for camrelizumab used in dMMR NECE cases, the diverse experience of camrelizumab application in other tumors are excellent source of case reference. An NCT04453930 study enrolled patients with ES-SCLC who had been administered IP plus camrelizumab and showed encouraging PFS and ORR [25]. Jiang et al. reported that an dMMR advanced gastric cancer patient received neoadjuvant therapy, including camrelizumab plus chemotherapy, which ultimately achieved pathologic complete response (pCR) without serious adverse effects [26]. A randomized phase 2 clinical trial conducted by Lei et al. found that the pCR rate of patients with resectable advanced non-small cell lung cancer (NSCLC) treated with camrelizumab combined with platinum-based chemotherapy vs. chemotherapy alone was 32.6% (95% CI, 19.1%–48.5%) and 8.9% (95% CI, 2.5%–21.2%), respectively [27]. The Major Pathological Response (MPR) rates of camrelizumab plus platinum-based chemotherapy were 65.1% (95% CI, 49.1%–79.0%) vs 15.6% (95% CI, 6.5%–29.5%) with chemotherapy alone. Based on the above research, camrelizumab combined with chemotherapy is a promising prospect in the treatment of dMMR cancers. Equally encouraging is this case of dMMR NECE treated with the combination therapy of camrelizumab and chemotherapy, which achieved the transformation from postoperative progression and metastasis to clinical complete response (cCR) following clinical assessment. An open-label prospective pivotal trial enrolled 12 patients with dMMR advanced or metastatic solid tumours who received camrelizumab treatment (200 mg every 2 weeks). The result demonstrated that 8 patients achieved ORR, with a disease control rate of 100%, a 12-month PFS rate of 83.3%, and an overall survival rate of 90% [28]. Monotherapy with camrelizumab for 1 year after combined therapy, our patient still maintains cCR. Our patient has shown two years of a durable and safe response for this time. During the camrelizumab therapy, the common adverse event is reactive cutaneous capillary endothelial proliferation [29]. However, the adverse effect was not observed in our patient. In summary, the addition of camrelizumab to chemotherapy may be considered as a potential therapeutic strategy for dMMR NECE, supported by diverse case studies. For such a cause, mismatch repair (MMR) or microsatellite instability (MSI) tests are essential for guiding treatment and survival in patients with NECE.

Related literature reports that postoperative adjuvant radiotherapy is helpful in improving the prognosis of NECE patients. Tu et al. reported 7 patients with NECE who received adjuvant pelvic radiotherapy. 3 patients survived for more than 12 months, and 4 lived without disease relapse [30]. Furthermore, Hu et al. reported a patient with NECE stage III C2, who was administered an EP regimen for 3 weeks at a time after curative resection. After 3 cycles of chemotherapy, the patient received local radiotherapy, and the reexamination displayed no evidence of further disease progression. Finally, the patient achieved a complete response [31]. Horeweg et al. collected data from the PORTEC-1 trial and the PORTEC-2 trial, with a total of 880 EC patients, and used Kaplan-Meier’s methodology and log-rank tests to compare the survival rate of pelvic radiotherapy with no adjuvant therapy. The result revealed in dMMR EC, the locoregional recurrence-free survival of radiotherapy was higher than that of no adjuvant therapy [32]. A retrospective analysis from a single center reported that 3 NECE patients received adjuvant radiotherapy after surgery, none of them experienced recurrence, and they achieved complete remission [33]. Therefore, adjuvant radiotherapy might have promising effects on NECE patients. Regarding the timing of adjuvant radiotherapy, based on the NCCN guidelines and the specific circumstances of this case, patients with advanced NECE should prioritize completing the planned systemic adjuvant therapy. Radiotherapy should be initiated within 4–8 weeks after achieving cCR, while concurrently avoiding the peak period of systemic treatment-related toxicities (such as bone marrow suppression). Initiating radiotherapy too early may increase radiotherapy-related hematologic toxicity due to incomplete recovery from chemotherapy-induced bone marrow suppression. Conversely, delaying treatment may miss the optimal window for local control, thereby elevating the risk of recurrence.

During the course of treatment, our patient developed radiotherapy-related advanced pelvic bone damage (pelvic insufficiency fracture, PIF) after radiotherapy. It may be related to the following 3 aspects. First, the direct action of radiation on the skeleton may cause a series of pathophysiologic changes in the skeletal system. Such as reducing the blood supply to the bones, decreasing the number and activity of osteoblasts, inducing apoptosis of osteoblasts, and decreasing bone mineral density [34]. Secondly, radiation may act on gonadal targets *in vivo*, leading to central or peripheral hypogonadism, inducing an imbalance in metabolic hormone levels in bones [35,36]. Thirdly, insufficient nutrition and lack of appropriate functional exercise in the body after radiotherapy can also affect the volume, weight-bearing, shaping, and reconstruction of bones. Sapienza et al. conducted a meta-regression study of 3929 gynecologic oncology patients who underwent pelvic radiotherapy. They found a 14% incidence of PIF, a median time to fracture ranging from 7.1 to 19 months after radiotherapy, and 61% patients had comorbid pain symptoms. In terms of the sites of PIF, sacrum and pubis accounted for 73.6% and 13%, respectively [37]. Razavian et al. reported a meta-analysis of 2131 patients; the 5-year actuarial incidence of PIF was 15.3% (95% CI 7.5%–25.0%), the incidence of PIF was 60.3% in the sacrum, 12.9% in the pubic bone, and 6% in the lumbar spine [38]. Ramlov et al. performed research based on radio-chemotherapy data of gynecological tumors; they found that age was a significant risk factor, and the incidence of PIF in patients ≤50 years old was significantly lower than that in patients >50 years old [39]. Initially, we thought the cause of lumbosacral pain developed 7 months after the completion of radiotherapy in our patient was due to the patient’s history of lumbar spine disease. The lumbosacral pain gradually worsened, and associated ancillary tests suggested the presence of a newly occurring fracture. A bone puncture biopsy confirmed that the bone damage was not caused by primary disease metastasis. Combined with the Dose-Volume Histogram data from the patient’s radiotherapy plan, indicating left femoral head V30 = 8% and right femoral head V30 = 8%, we therefore concluded that this was a radiotherapy-induced PIF. About 50% of clinical PIF patients may be complicated with pain (some of them are manifested as severe pain, and require hospitalization due to decreased or lost mobility), while 40%–60% patients have no specific signs on physical examination [37–39]. This may lead to neglect by patients or omissions and misdiagnosis by doctors (most diagnoses are bone metastases) [40]. Accordingly, improving the diagnosis of PIF must fully incorporate the patient’s radiotherapy history, pelvic imaging, and bone puncture pathology biopsy if necessary. Additionally, for patients scheduled to undergo pelvic radiotherapy, comprehensive baseline bone assessments must be completed prior to treatment, including DEXA scans, vitamin D and calcium levels, and endocrine testing. Patients with benign bone lesions may receive calcium/vitamin D supplementation during treatment, and bone protectors may be administered as needed to prevent radiotherapy-related PIF.

Our patient, in this case, developed tumor recurrence with rapid progression, simultaneously accompanied by severe septic shock after the operation and the first cycle of EP. The tumor recurrence is likely related to the high malignancy of the tumor itself and incomplete resection. Generally, standard surgical procedures for endometrial malignancies include total hysterectomy, bilateral salpingo-oophorectomy, and lymph node dissection. Zhang and Pang reported 142 patients with advanced-stage NECE; the 5-year OS rates for surgery + concurrent chemoradiotherapy, surgery + chemotherapy, surgery alone, and chemotherapy alone were 65.27%, 17.76%, 7.69%, and 4.16%, respectively [41]. In a previous report, a retrospective and multi-institutional study in Japan had enrolled 42 cases of NECE from 18 medical institutions, the results showed that complete surgery improved the prognosis of advanced-stage NECE patients [42], suggesting that the surgery is recommended for patients who are eligible for surgery and for whom the tumor can be completely resected should undergo surgery, as they are likely to benefit from such surgical intervention. However, for our patient, further preoperative evaluation and multidisciplinary discussion and consultation were required to determine whether surgery was appropriate. Additionally, there should be a sufficient time interval between surgery and postoperative chemotherapy, generally at least two weeks, to allow for recovery. Chemotherapy administered too close after surgery is likely to result in severe chemotherapy-related adverse effects due to insufficient recovery, as in our case, the patient developed septic shock, which ultimately posed a life-threatening risk. Some reports have reported common adverse effects of EP, including leukopenia, neutropenia, and anemia caused by varying degrees of myelosuppression [43,44]. We believed that the infection was due to severe post-surgical trauma and the myelosuppression after chemotherapy. However, there is still no conclusive answer as to whether neoadjuvant chemotherapy before surgery or adjuvant chemotherapy after surgery is more effective due to the lack of data from the standard treatment for NECE advanced patients. Further research and exploration are needed to determine the optimal treatment strategy.

In summary, the current attempt to treat our patient with dMMR NECE IVB using camrelizumab combined with chemotherapy, followed by camrelizumab monotherapy for maintenance treatment and adjuvant radiotherapy regimen, is successful. We believe our study proposes a brand-new treatment approach for advanced dMMR NECEs, although due to the rare occurrence of dMMR NECE in the clinic, the cases that support our treatment strategy may be limited. Further investigations will be required to validate our findings.

## Supplementary Materials



## Data Availability

The original contributions presented in the study are included in the article/supplementary materials. Further inquiries can be directed to the corresponding author.

## Supplementary Materials

The supplementary material is available online at https://www.techscience.com/doi/10.32604/or.2026.071213/s1. Figure S1: Radiotherapy plan. (A) The local external-beam radiotherapy target volume. (B,C) Dose-Volume Histogram (DVH).
