# Supplementary material for: Case Report: A Case of Neuroendocrine Carcinoma of the Endometrium with Deficient DNA Mismatch Repair Had Achieved Clinical Complete Response after Combination Therapy
Source: Oncol Res. 2026 Apr 22;34(5):37. doi: 10.32604/or.2026.071213 (PMC13126403; doi:10.32604/or.2026.071213)
Supplement: Supplementary file 1 [file OncolRes-34-71213-s001.zip › OR_71213-s001/Checklist.docx]

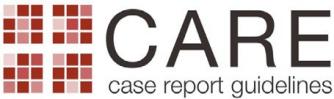

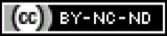

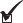
**CARE Checklist of information to include when writing a case report**

| **Topic** | **Item No** | **Checklist item description** | **Reported on Page Number/Line Number** | **Reported on Section/Paragraph** |
| --- | --- | --- | --- | --- |
| Title | 1 | The diagnosis or intervention of primary focus followed by the words “case report” |  | 1 |
| Key Words | 2 | 2 to 5 key words that identify diagnoses or interventions in this case report, including "case report" |  | 1 |
| Abstract  (Structured summary) | 3a | Background: state what is known and unknown; why the case report is unique and what it adds to existing literature. |  | 1 |
|  | 3b | Case Description: describe the patient’s demographic details, main symptoms, history, important clinical findings, the main diagnosis, interventions, outcomes and follow-ups. |  | 1 |
|  | 3c | Conclusions: summarize the main take-away lesson, clinical impact and potential implications. |  | 1 |
| Introduction | 4 | One or two paragraphs summarizing why this case is unique **(may include references)** |  | 1 |
| Patient Information | 5a | De-identified patient specific information |  | 2 |
|  | 5b | Primary concerns and symptoms of the patient |  | 2 |
|  | 5c | Medical, family, and psycho-social history including relevant genetic information |  | 2 |
|  | 5d | Relevant past interventions with outcomes |  | 2 |
| Clinical Findings | 6 | Describe significant physical examination (PE) and important clinical findings |  | 2 |
| Timeline | 7 | Historical and current information from this episode of care organized as a timeline |  | 2 |
| Diagnostic Assessment | 8a | Diagnostic testing (such as PE, laboratory testing, imaging, surveys). |  | 2 |
|  | 8b | Diagnostic challenges (such as access to testing, financial, or cultural) |  | 2 |
|  | 8c | Diagnosis (including other diagnoses considered) |  | 2 |
|  | 8d | Prognosis (such as staging in oncology) where applicable |  | 2 |
| Therapeutic Intervention | 9a | Types of therapeutic intervention (such as pharmacologic, surgical, preventive, self-care) |  | 2 |
|  | 9b | Administration of therapeutic intervention (such as dosage, strength, duration) |  | 2 |
|  | 9c | Changes in therapeutic intervention (with rationale) |  | 2 |

| Follow-up and Outcomes | 10a | Clinician and patient-assessed outcomes (if available) |  | 2 |
| --- | --- | --- | --- | --- |
|  | 10b | Important follow-up diagnostic and other test results |  | 2 |
|  | 10c | Intervention adherence and tolerability (How was this assessed?) |  | 2 |
|  | 10d | Adverse and unanticipated events |  | 2 |
| Discussion | 11a | A scientific discussion of the strengths AND limitations associated with this case report |  | 3 |
|  | 11b | Discussion of the relevant medical literature **with references** |  | 3 |
|  | 11c | The scientific rationale for any conclusions (including assessment of possible causes) |  | 3 |
|  | 11d | The primary “take-away” lessons of this case report (without references) in a one paragraph conclusion |  | 3 |
| Patient Perspective | 12 | The patient should share their perspective in one to two paragraphs on the treatment(s) they received |  |  |
| Informed Consent | 13 | Did the patient give informed consent? Please provide if requested | **Yes √** | **No** |

*As the checklist was provided upon initial submission, the page number/line number reported may be changed due to copyediting and may not be referable in the published version. In this case, the section/paragraph may be used as an alternative reference.
