# Supplementary figures and images for: Case Report: A Case of Neuroendocrine Carcinoma of the Endometrium with Deficient DNA Mismatch Repair Had Achieved Clinical Complete Response after Combination Therapy
Source: Oncol Res. 2026 Apr 22;34(5):37. doi: 10.32604/or.2026.071213 (PMC13126403; doi:10.32604/or.2026.071213)

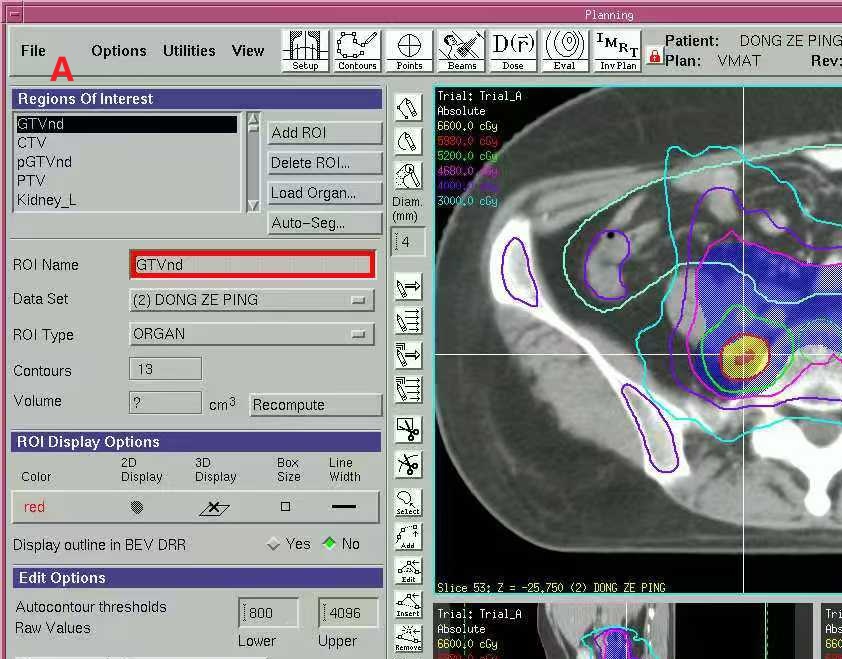

Supplement: Supplementary file 1 [file OncolRes-34-71213-s001.zip › OR_71213-s001/Figure S1/Supplementary Files/Supplementary Figure S1 A.TiF]

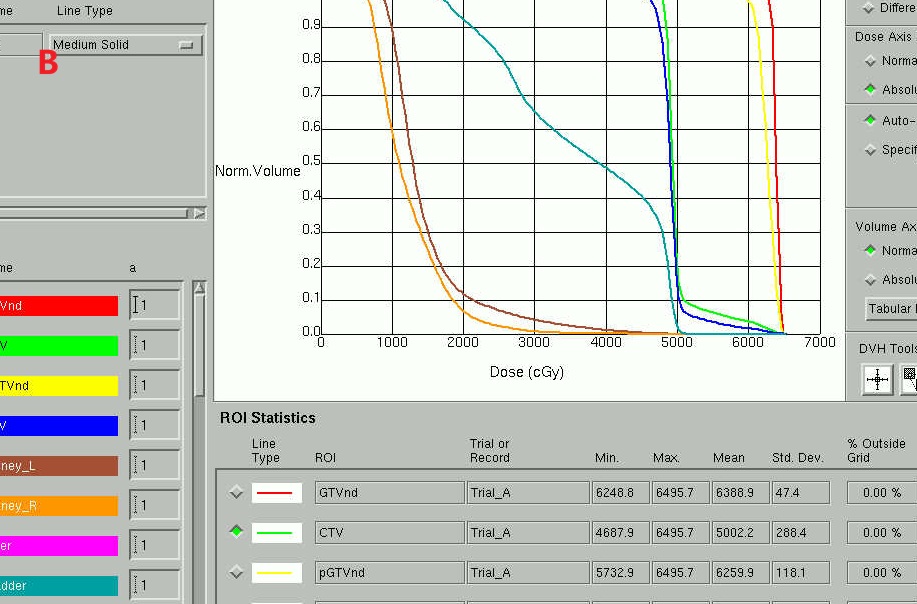

Supplement: Supplementary file 1 [file OncolRes-34-71213-s001.zip › OR_71213-s001/Figure S1/Supplementary Files/Supplementary Figure S1 B.Tif]

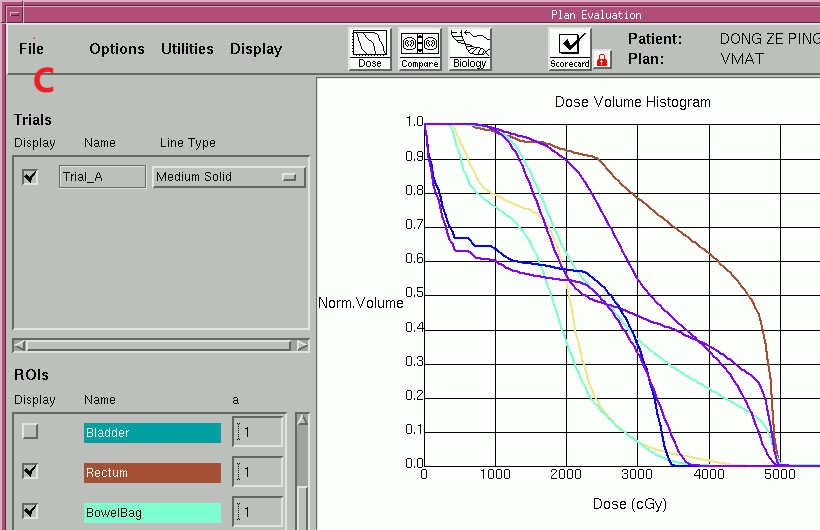

Supplement: Supplementary file 1 [file OncolRes-34-71213-s001.zip › OR_71213-s001/Figure S1/Supplementary Files/Supplementary Figure S1 C.Tif]
